# Machine learning unravels the mysteries of glioma typing and treatment

**DOI:** 10.1016/j.bbrep.2025.101969

**Published:** 2025-03-07

**Authors:** Ying Dang, Youhu Chen, Jie Chen, Guoqiang Yuan, Yawen Pan

**Affiliations:** a The Second Medical College of Lanzhou University, Lanzhou, Gansu, 730030, PR China; b Department of Neurosurgery, Second Hospital of Lanzhou University, Lanzhou, Gansu, 730030, PR China; c Key Laboratory of Neurology of Gansu Province, Lanzhou University. Lanzhou, Gansu, 730030, PR China; d Xijing Hospital, Air Force Medical University, Xi'an, Shaanxi Province, 710032, PR China; e The Northern Medical District, Chinese PLA General Hospital, Beijing, 100089, PR China

**Keywords:** Gliomas, WGCNA, Machine learning, Lasso. RF, SVM

## Abstract

Gliomas, which are complex primary malignant brain tumors known for their heterogeneous and invasive nature, present substantial challenges for both treatment and prognosis. Recent advancements in whole-genome studies have opened new avenues for investigating glioma mechanisms and therapies. Through single-cell analysis, we identified a specific cluster of cancer cell-related genes within gliomas. By leveraging diverse datasets and employing non-negative matrix factorization (NMF), we developed a glioma subtyping method grounded in this identified gene set. Our exploration delved into the clinical implications and underlying regulatory frameworks of the newly defined subtype classification, revealing its intimate ties to glioma malignancy and prognostic outcomes. Comparative assessments between the identified subtypes revealed differences in clinical features, immune modulation, and the tumor microenvironment (TME). Using tools such as the *limma* R package, weighted gene co-expression network analysis (WGCNA), machine learning methodologies, survival analyses, and protein-protein interaction (PPI) networks, we identified key driver genes influencing subtype differentiation while quantifying associated outcomes. This study not only sheds light on the biological mechanisms within gliomas but also paves the way for precise molecular targeted therapies within this intricate disease landscape.

## Highlights

• Single-cell analysis identifies malignant gene clusters in glioma.
• Prognostic subtypes of gliomas constructed & clinical characteristics analyzed.
• Explore subtype mechanisms via GO, KEGG, TME, immune analysis & Hallmark genes.
• Identify subtype-specific prognostic genes & establish scoring system.
• Evaluate immunotherapy efficacy & core gene expression across cancers.

## 1 Introduction

Gliomas, which are the most common malignancies that originate from neuroepithelial cells in the central nervous system (CNS), pose considerable clinical difficulties [1]. Gliomas make up almost 70 % of brain tumors [2], and they have a high global incidence rate, with an estimated 46 cases per 100,000 individuals each year [3], resulting in up to 30,000 deaths annually. Despite improvements in therapy, individuals diagnosed with glioblastoma multiforme (GBM), the most aggressive form of glioma, nevertheless have a bleak outlook. The average survival time for GBM patients is limited to 12–15 months, with less than 5 % of patients surviving for 5 years or more [4]. While surgery, radiotherapy, and radiochemotherapy are the primary methods used to treat glioma, the median overall survival (OS) for GBM patients is only 14–17 months [5]. Therefore, it is crucial to prioritize the discovery of viable therapeutic targets.

Since 2007, the classification of glioma has been significantly transformed to include molecular diagnostics [6]. The existing categorization approach integrates essential molecular indicators, including isocitrate dehydrogenase (*IDH*) mutations, 1p/19q codeletion, *ATRX* and/or *TP53* mutations, and promoter methylation of the methyl-guanine methyl transferase (*MGMT*) gene, thereby enhancing the accuracy of the diagnostic procedure [7]. Importantly, the status of *IDH* is a crucial indicator for categorizing gliomas, where all gliomas without *IDH* mutations are categorized as grade 4 according to the World Health Organization (WHO) classification for central nervous system (CNS) tumors. This finding emphasizes the diagnostic and prognostic importance of *IDH* status. Although there have been significant advancements in this field [8,9], it is still difficult to discover effective therapeutic targets. Therefore, further research on the molecular changes that cause gliomagenesis is needed.

The emergence of bioinformatics and functional genomics has enabled the creation of important databases, such as the Gene Expression Omnibus (GEO), The Cancer Genome Atlas (TCGA), and the Chinese Glioma Genome Atlas (CGGA)[[10], [11], [12]]. They offer researchers vital resources for identifying possible oncogenic drivers and accelerating the discovery of molecular targets involved in glioma formation.

Cancer may originate from gene mutations and abnormal gene expression. To examine the molecular mechanisms that are responsible for cancer-related gene sets in gliomas, we employed single-cell analysis to identify gene sets that are specifically associated with tumors. Through the integration of several glioma datasets, we utilized non-negative matrix factorization (NMF) to generate glioma prognostic subtypes using these cancer gene sets. Two distinct subtypes were identified: Glioma-associated Malignant Cell-Infiltrative (GMC-I) and Glioma-associated Malignant Cell-Non-Infiltrative (GMC-NI). Significantly, patients with the GMC-NI subtype exhibited a better prognosis than those with the GMC-I subtype. We evaluated clinical factors such as tumor grade, patient sex, patient age, *IDH* mutation status, 1p19q mutation status, *MGMT* promoter (*MGMT*p) methylation status, immune microenvironment status, and tumor mutation burden (TMB). Our data indicated that the GMC-I subtype exhibited elevated tumor purity, a greater number of gene mutations, a greater percentage of high-grade gliomas and older patients, and a greater frequency of *IDH* wild-type and 1p19q codeletion.

Using hallmark gene sets, gene set enrichment analysis (GSEA) revealed upregulated expression of EPITHELIAL_MESENCHYMAL_TRANSITION, TNFA_SIGNALING_VIA_NFKB, and KRAS_SIGNALING_UP in the GMC-I subtype. This suggests that the poor prognosis of patients with the GMC-I subtype may be attributed to aberrant abnormal tumor proliferation. Previous research has revealed that epithelial-mesenchymal transition (EMT), which is linked to the rapid growth and spread of tumors, promotes the formation of malignant tumors and is associated with a negative prognosis. These characteristics coincide with our observations of the GMC-I subtype. Disease enrichment analysis demonstrated that the differentially expressed genes (DEGs) between the two subtypes were associated with a range of malignancies, including high-grade gliomas. The immune analysis showed that the GMC-I subtype had a greater percentage of M2 macrophages and had significant immunosuppressive effects. Using the *limma* R package and the weighted gene co-expression network analysis (WGCNA) algorithm, we identified driver genes that differentiated the two subtypes. By employing prognostic analysis, machine learning, and protein‒protein interaction (PPI) analysis, we determined that *CD47*, *CTSZ*, and *SLC11A1* are driver genes affecting the outcomes of different subtypes. Next, we quantified the two subtypes using the single-sample GSEA (ssGSEA) algorithm and confirmed the accuracy of our findings by comparing them with external datasets. Immunotherapy predictions indicated that the GMC-NI subtype responded better to treatment.

In summary, we constructed molecular subtypes of gliomas and examined the inherent relationships and distinctions between the two subtypes in relation to clinical traits, molecular characteristics, immune functions, oncogenic pathways, TMB, and the tumor microenvironment (TME). Our findings revealed potential molecular pathways that contribute to the classification and prognosis of glioma, offering new opportunities for further research on the molecular classification of gliomas.

## 2 Materials and methods

### 2.1 Data sources

The single-cell dataset was obtained from the Gene Expression Omnibus (GEO) database (https://www.ncbi.nlm.nih.gov/geo/) under accession number GSE182109. Transcriptome expression data and clinical data were derived from two sources: the Chinese Glioma Genome Atlas (CGGA) database (http://www.cgga.org.cn) and The Cancer Genome Atlas (TCGA) database (https://portal.gdc.cancer.gov) [[13], [14], [15], [16], [17], [18]]. We use the limma [19,20] and sva R packages to identify commonly differentially expressed genes between the two datasets use the rbind function to merge the datasets and apply the ComBat function to perform batch correction on the combined dataset.

### 2.2 Single-cell analysis for identifying tumor subtypes

The GSE182109 dataset was retrieved from the GEO at the National Center for Biotechnology Information (NCBI) (https://www.ncbi.nlm.nih.gov/geo/) and processed using the Seurat R package (version 4.3.0) for quality control (QC) and subsequent functional analysis. Cells of inferior quality were excluded. The LogNormalize function was used for data normalization by scaling the expression levels of each feature based on the overall expression in each cell. The FindVariableFeatures function was applied to identify variable features, typically with 2000 features selected by default. Principal component analysis (PCA) was conducted for dimensionality reduction, and then graph-based clustering was performed in the PCA space for cluster analysis. Clusters were visualized using t-distributed stochastic neighbor embedding (t-SNE). Additionally, Seurat functions, including Heatmap, FeaturePlot, VlnPlot, and DotPlot, were utilized for gene expression visualization. Furthermore, the FindAllMarkers function was used to find markers distinct to certain clusters in comparison to all other clusters.

### 2.3 Subtype identification and clinical Correlation Analysis

We applied NMF to a gene set consisting of 1357 tumor cell genes to identify subtypes and evaluate their associations with clinical phenotypes. Differential expression analysis was conducted using the *limma* package, resulting in DEGs, followed by Disease Ontology (DO) analysis to investigate the activation status of related tumors. Subsequently, we utilized the Gene Ontology (GO) database to assess molecular function (MF), biological process (BP), and cellular component (CC) terms. Kyoto Encyclopedia of Genes and Genomes (KEGG) analysis and specific gene set analysis were also performed.

### 2.4 WGCNA

We used the WGCNA method to find modules linked to two clusters [21], with the soft-thresholding power determined using the scale-free topology criterion to find the best value. A minimal module size of 50 genes was set, and modules were identified using the dynamic tree cut method with a MEDissThres value of 0.25.

### 2.5 Acquisition of gene intersections and construction of a machine learning-based joint model for gene identification

Machine learning, which enables robots to learn and detect patterns from complex data, was employed to predict future outcomes and trends [22]. Initially, differential analysis of the two subtypes revealed 206 DEGs. By intersecting these genes with the 252 module-related genes identified through WGCNA, 180 DEGs associated with specific features were obtained. Subsequently, using the R packages *survival* and *survminer* with a coxPfilter threshold of 0.05, the iterative analysis identified 924 genes related to prognosis. After intersecting these genes, a total of 180 common genes were obtained.

We employed three efficient machine learning algorithms—Random Forest, LASSO Regression, and SVM—to accurately identify core genes. Random Forest effectively improves model accuracy and stability by integrating multiple decision trees, excelling in handling high-dimensional data, robustly resisting overfitting, and providing feature importance evaluation [23]. LASSO Regression utilizes the L1 regularization mechanism to achieve feature selection, automatically screening out key features, reducing model complexity, and enhancing generalization ability, making it particularly suitable for processing high-dimensional and noisy data. SVM, on the other hand, performs well in handling high-dimensional and nonlinear data, accurately identifying core genes by finding the optimal decision boundary and assessing the importance of each feature through analysis of feature weights. Random Forest has unique advantages in high-dimensional data processing and overfitting resistance [24], LASSO Regression possesses powerful feature selection capabilities, and SVM can effectively address nonlinear problems. These three algorithms complement each other, providing a comprehensive and effective solution for identifying core genes, which helps us understand gene expression data more accurately and reveal the core mechanisms of disease occurrence and development. LASSO, RF, and SVM were employed to pinpoint significant genes, resulting in the identification of 17 key genes. Finally, the study of the PPI network highlighted three core genes.

### 2.6 Prognostic validation and immunotherapy assessment

The ssGSEA algorithm was used to obtain sample scores for the three key genes, and the samples were then categorized into high- and low-risk subgroups based on the median value. The TCGA dataset and the CCGA mRNA-array_301 dataset were utilized for prognostic validation, We obtained transcriptome data of immune-related genes following PD-1 blockade from the IMvigor210 dataset for immunotherapy evaluation in this study.

### 2.7 Statistical analysis

Data analysis was conducted using R version 4.3.2 (available at http://www.r-project.org). The Wilcoxon test was used to compare continuous data between the two subtypes, while the Pearson correlation coefficient was used to evaluate associations between them. K‒M survival analysis was employed to assess and compare survival outcomes in the two subtypes, with statistical significance defined as a *p-value* less than 0.05.

## 3 Results

### 3.1 Acquisition of cancer cell subtypes

We retrieved the GSE182019 dataset from the GEO database and extracted samples for t-SNE sample clustering (Fig. 1A). Following batch correction using Harmony (Fig. 1B–C), an appropriate number of principal components (PCs) (PC = 20) was selected (Fig. 1D–F). Using singleR and manual annotation based on marker genes, we classified cells into four subpopulations: immune cells (identified by markers CD14, FCGR1A, MSR1, and CD163), oligodendrocytes (identified by markers OLG2, FA2H, UGT8, and CNP), astrocytes (identified by marker GFAP), and cancer cells (identified by markers PARP1, CD44, and IFAP) (Fig. 1G–I). Subsequently, genes related to the cancer cell subpopulation were extracted (Table S1).

**Fig. 1:**
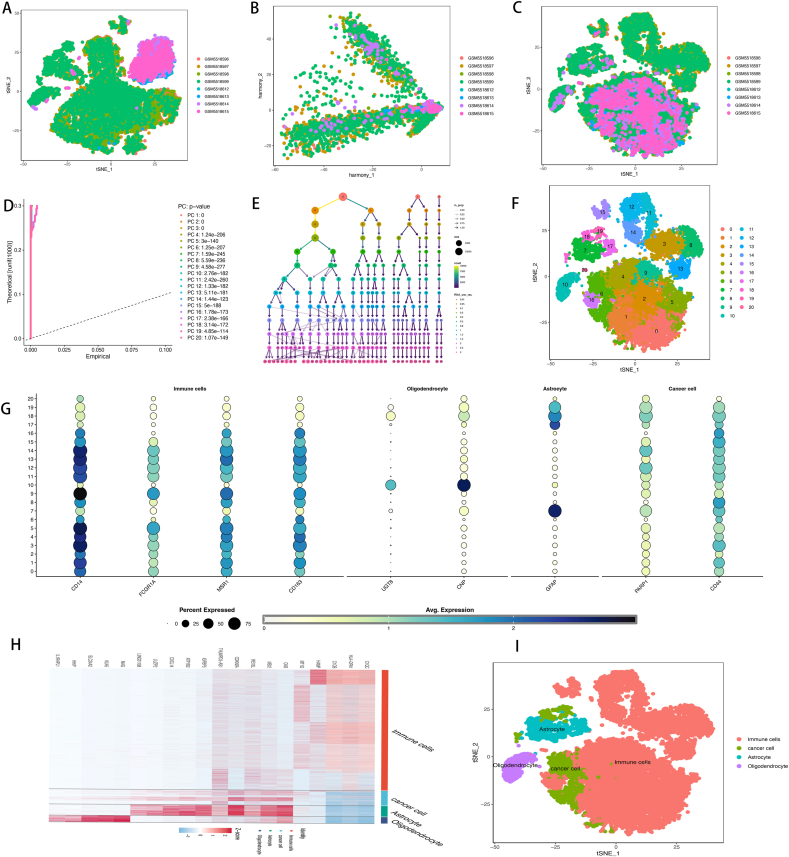
Identification of Tumor Subpopulations through Single-Cell Analysis. **A.***t-SNE Clustering of Samples; B. Batch Effect Removal Using Harmony; C. Display of Clusters After Batch Effect Removal; D-E. Selection of Principal Components (PCs); F. Clustering Based on PCs; G-I. Cluster Identification Using Marker Genes.*

### 3.2 Subtype acquisition and clinical Correlation Analysis

**Fig. 2:**
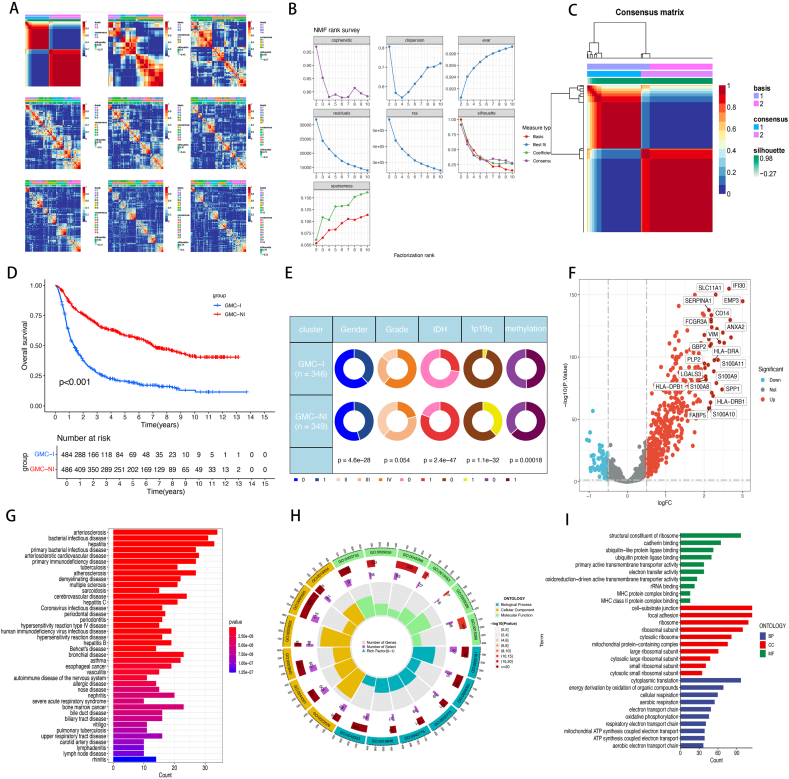
Acquisition of subtypes and clinical characteristics.

The CGGA database provides a comprehensive dataset comprising 1018 samples, including two subsets, CGGA325 and CGGA693. To eliminate batch effects, we merged these two datasets and performed batch correction ((Figs. S1A–S1B). Subsequently, we extracted genes closely related to cancer cell subpopulations from the dataset. To assess the distribution and clustering of these genes among the samples, we applied Non-negative Matrix Factorization (NMF) to the transcriptome data of the 1018 samples and evaluated the grouping quality from 2 to 10 groups ((Fig. 2A–B). During this process, we excluded samples with survival times less than 0, ultimately retaining 970 samples for subsequent analysis. The optimal clustering was achieved in two distinct transcriptional categories, designated GMC-I and GMC-NI (Fig. 2C). Clinical trait analysis indicated that high-grade glioma infiltration was more frequent in the GMC-I subtype, and there was also an increase in infiltration in older patients and those with IDH wild-type glioma in this subtype. The 1p19q non-co deleted status and *MGMT*p_un-methylated conditions were also enriched in the GMC-I subtype. Additionally, high-grade gliomas were more frequent in the high infiltration group (Fig. 2D). Prognostic assessment indicated a poorer prognosis for the GMC-I subtype than for the GMC-NI subtype (Fig. 2E). Differential analysis identified 206 DEGs (Fig. 2F), and disease enrichment analysis linked these genes to various tumors, including high-grade gliomas. GO analysis suggested associations with major histocompatibility complex (MHC) and ribosome function, indicating proliferation and immune-related characteristics (Fig. 2G–I).

A-B. Subtype Heatmap and NMF Rank Survey; C. Binary Subtype Heatmap; D. Survival Analysis of Subtypes; E. Circle Plot of Subtypes and Clinical Characteristics; F. Volcano Plot of Differential Genes Between Subtypes; G. Disease Enrichment Analysis of Differential Gene Sets; H–I. GO Analysis of Differential Genes.

### 3.3 Subgroup immune landscape suggests immunosuppression in the GMC-I subgroup

**Fig. 3:**
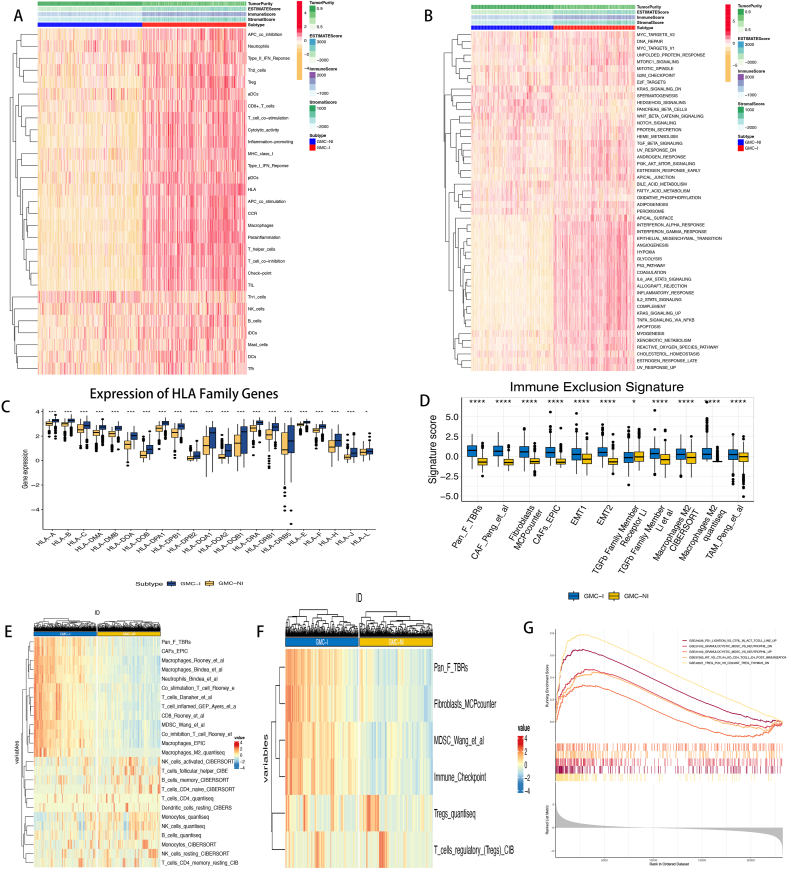
Subpopulation immune and pathway landscape. *A. Tumor Microenvironment, Immune Function, and Gene Enrichment; B. Distribution Landscape of Hallmark Gene Sets; C–F. Distribution Landscape of Immune Cells.*

The change in the proportion of immune cells and stromal cells in the tumor microenvironment (TME) is one of the core mechanisms driving tumor initiation and progression. We conducted an in-depth and comprehensive analysis of the TME in different subgroups. In the GMC-I subtype, the proportion of stromal cells was significantly higher than that in the GMC-NI subtype, and the tumor purity was relatively lower (Fig. 3A). This change was accompanied by the abnormal activation of multiple immunosuppressive functions and pathways.

Through Hallmark gene set enrichment analysis, we found abnormal activation of epithelial-mesenchymal transition (EMT) and activation of multiple oncogenic pathways, including the P53 pathway and IL6_JAK_STAT3_SIGNALING, in the GMC-I subtype (Fig. 3B). These abnormally activated pathways may be one of the intrinsic molecular mechanisms leading to excessive proliferation of tumors in the GMC-I subtype, thereby mediating the poor prognosis of gliomas.

In addition, we investigated the expression differences of human leukocyte antigen (HLA) family genes between the two subgroups (Fig. 3C). The results showed that all HLA family genes were highly expressed in the GMC-I subtype with poor prognosis. Further analysis of the immune rejection score revealed that the immune rejection score was significantly higher in the GMC-I subtype than in the GMC-NI subtype (Fig. 3D), indicating that the GMC-I subtype may be insensitive to immunotherapy, while the GMC-NI subtype is more likely to benefit from immunotherapy.

We also assessed the distribution of immune cell types and immunosuppressive signals in the TME of patients with GMC-I and GMC-NI subtypes (Fig. 3E and F). The results showed that cancer-associated fibroblasts (CAFs), myeloid-derived suppressor cells (MDSCs), M2 macrophages, and immune checkpoints were significantly enriched in the GMC-I subtype, which was closely related to its poor prognosis. To gain a deeper understanding of the immune status in the GMC-I subtype, we downloaded immunologic signature gene sets closely related to immune cell function, immune escape, and inflammatory response from a molecular tag database. Through gene set enrichment analysis (GSEA), we evaluated the activation status of these pathways in the GMC-I subtype (Fig. 3G). The results showed that immunosuppression-related pathways, including the CTLA-4/B7 pathway, PD-1/PD-L1 pathway, MDSCs, and regulatory T cell (Treg)-mediated immunosuppression pathway, were activated in the GMC-I subtype.

Furthermore, we performed an analysis of 22 types of immune cell infiltration between the two subtypes. The results indicated that the infiltration of M2 macrophages was higher in the GMC-I subtype (Fig. S1C). These findings suggest that glioma patients with characteristics of the GMC-NI subtype are more likely to be classified as “hot immune tumors.” Molecular markers related to immunosuppression and rejection, such as the EMT pathway, are also mainly enriched in the GMC-I subtype, indicating an immunosuppressive state. This means that the GMC-I subtype is more inclined to be a “cold immune tumor."

### 3.4 WGCNA network module mining

**Fig. 4:**
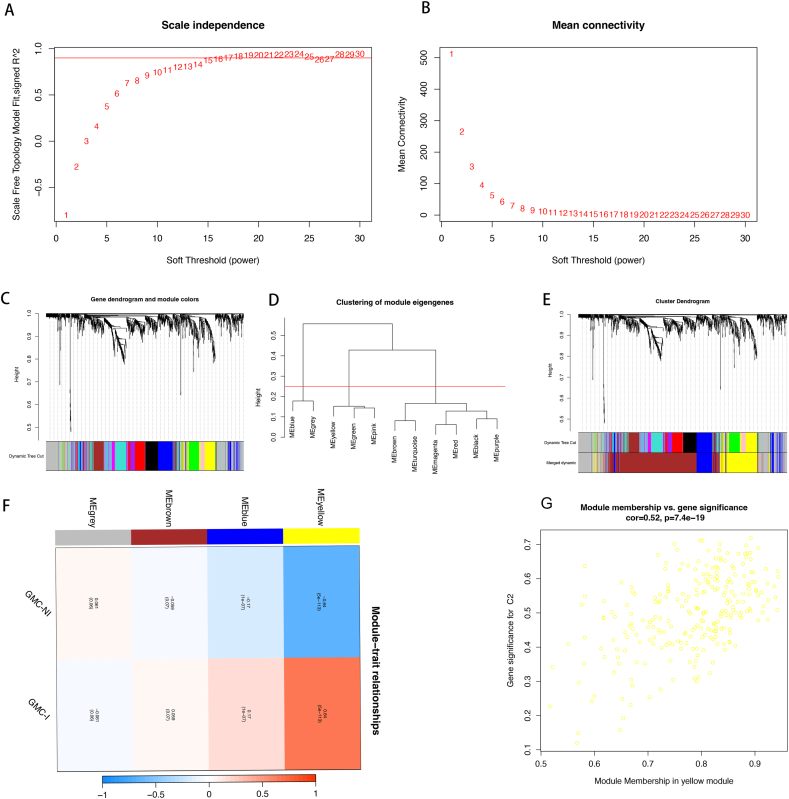
WGCNA Analysis of Typing *A. Confirmation of the Optimal Scale-Free Index (β) and Average; connectivity for Various Soft Threshold Powers; B. Gene Tree Diagram and Module Colors; C. Hierarchical Cluster Analysis; D. Gene Tree Based on Clustering Results; E. Heat Map of the Correlation Between Module Genes and the Two Isotypes; F. Correlation Between Genes and Subgroup* GMC-NI (C2).

The scale-free topology criterion was used to determine the soft-thresholding value. Subsequently, based on the phylogenetic tree, a soft-thresholding power of β = 14 was identified (Fig. 4A–B) for the identification of co-expression modules with a cutting height ≥0.25. Hierarchical clustering of modules within the same branch revealed similar gene expression patterns (Fig. 4C–E). Therefore, these similar gene modules were grouped together to form four co-expression modules, represented as gray, brown, blue, and yellow (Fig. 4G). Visualization of the gene clusters and analysis of the correlations between modules indicated a strong correlation between the yellow module and the GMC-I subtype (Fig. 4F–G). This finding prompted further investigation (Table S2).

### 3.5 Dimensionality reduction and machine learning analysis

**Fig. 5:**
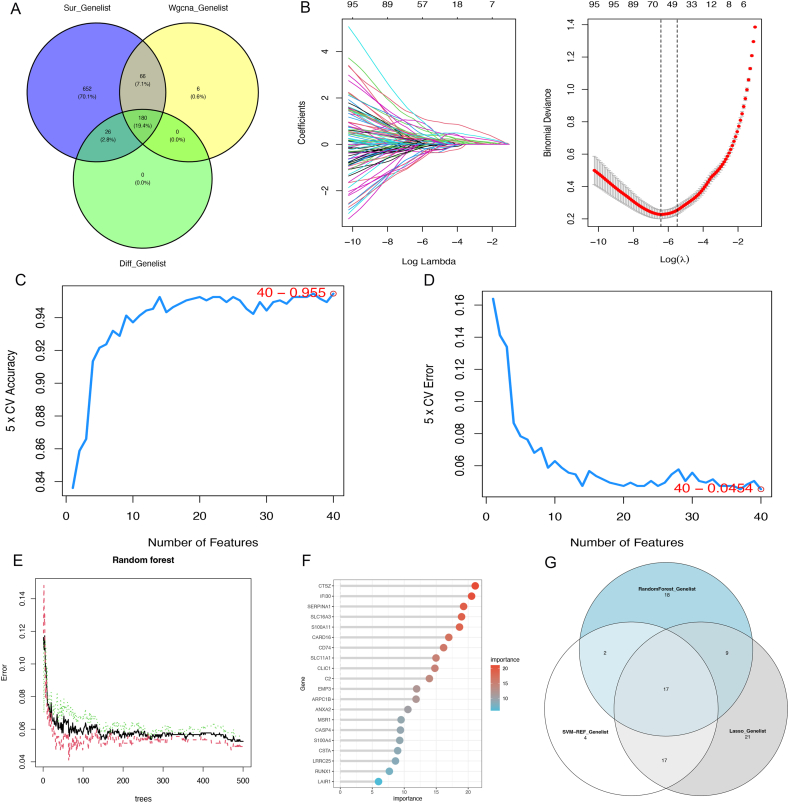
Identification of core genes using machine learning. *A. Intersection of Module Genes, Differential Genes, and Survival Genes; B. Lasso Regression Analysis; C-D. Support Vector Machine Analysis; E-F. Random Forest Analysis; G. Intersection of Genes Identified by Three Machine Learning Methods.*

We identified 252 module-related genes and 206 DEGs(Table S3). Using *survival* and *survminer* packages, we identified 180 survival-related genes that were strongly associated with the GMC-I subtype (Fig. 5A). Using these 180 survival-related genes, we conducted analyses using LASSO regression, RF, and SVM. In the LASSO regression analysis, 64 genes were selected (Fig. 5B), while the SVM analysis identified 40 genes (Fig. 5C–D), and the RF analysis selected 46 genes (Fig. 5E–F). According to the results of these analyses, we identified a total of 17 core genes (Fig. 5G).

### 3.6 Subgroup quantification and immunotherapy prediction

**Fig. 6:**
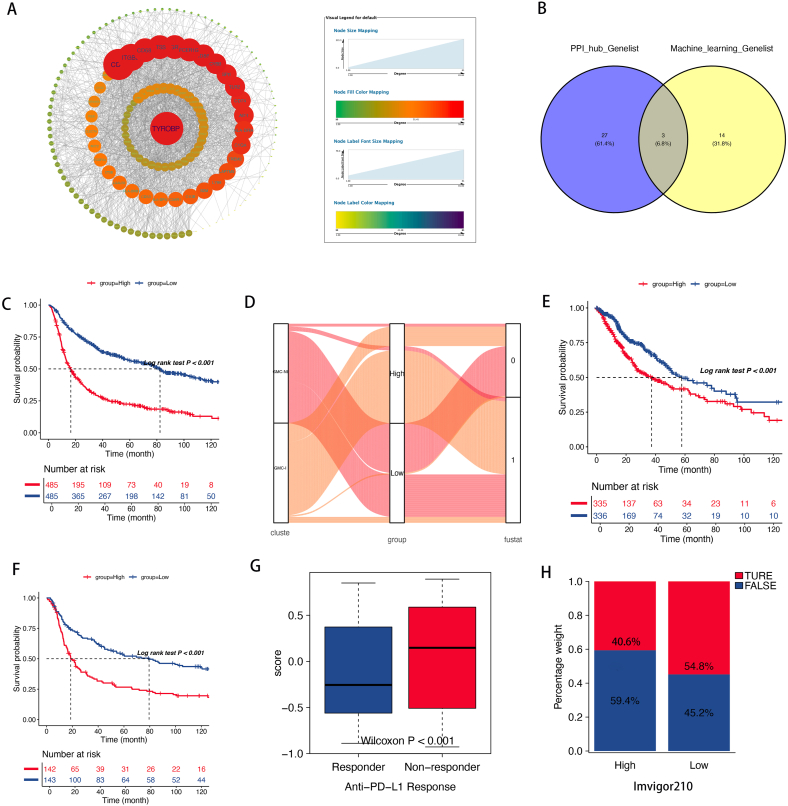
Quantification of subtypes and prediction of immunotherapy response. *A. Protein-Protein Interaction Network Analysis; B. Intersection of Protein-Protein Interaction Genes and Machine Learning-Identified Genes; C. Survival Display After Quantification; D. Sankey Plot of Subtypes and Quantification Results; E. TCGA Validation Set; F. CGGA301 Validation Set; G-H. Prediction of Immunotherapy Response.*

A PPI network analysis was performed on the initial gene set to identify key genes. Using the STRING database to obtain protein nodes, we visualized the genes using Cytoscape (Fig. 6A). After intersecting with the core genes, we identified three key genes: CD47, CTSZ, and SLC11A1 (Fig. 6B). Subsequently, we utilized the ssGSEA algorithm to obtain sample scores for the core genes, quantifying our samples into high- and low-risk groups showing significant prognostic differences (Fig. 6C). Sankey diagram visualization indicated low-risk characteristics for GMC-I and high-risk characteristics for GMC-NI, with samples from both groups being almost completely concordant (Fig. 6D). We further validated our analysis results across the TCGA and CCGA mRNA-array_301 datasets (Fig. 6E–F). Immunotherapy produced a better response in the low-risk group, which is consistent with our previous findings (Fig. 6G–H).

### 3.7 Key genes exhibiting cancer cell characteristics

**Fig. 7:**
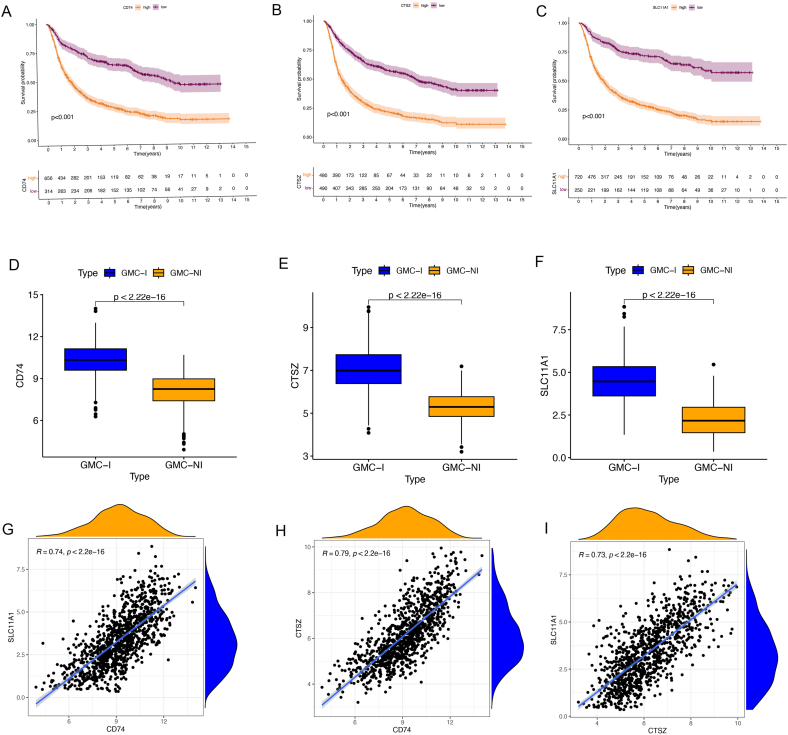
Core gene Analysis. A-C. Survival analysis; D-F. Subtype expression analysis; G-I. Correlation analysis of core genes.

We analyzed the core genes within our dataset and found that high expression levels of CD47, CTSZ, and SLC11A1 were associated with poor prognosis (Fig. 7A–C). Upon examining the expression of these key genes within the two subgroups, we found that all three genes were highly expressed in the GMC-I subtype, which is the group with a poor prognosis (Fig. 7D–F). We conducted an ROC curve analysis to evaluate the performance of core genes in subtype prediction. The analysis results showed that the AUC values of all core genes exceeded the threshold of 0.9, fully demonstrating their excellent classification and prediction capabilities (Fig. S1 D-F). Using the rms package, we developed a nomogram model for the diagnosis of the GMC-I subtype based on the biomarker genes CD47, CTSZ, and SLC11A1 (Fig. S1 G).

**Fig. 8:**
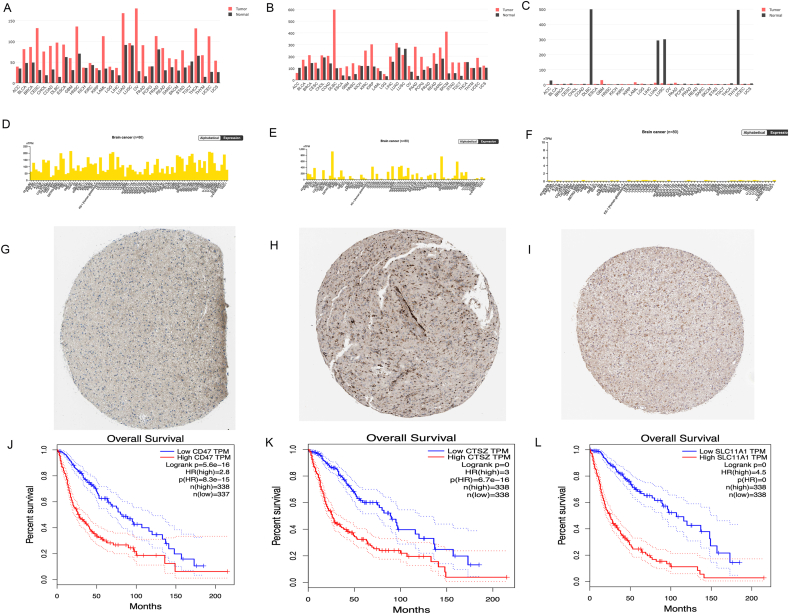
Oncogenic Properties of core Genes. A-C. Expression profiles of core genes in normal and tumor tissues across 33 cancer types; D-F. Expression of core genes in Glioma cell lines; G-I. Immunohistochemistry results showing high expression of core genes in Glioma patients; J-K. High expression of core genes in TCGA data mediating poor prognosis.

Additionally, there were significant correlations among these three genes (R > 0.73, *P* < 2.2e-16) (Fig. 7G–I). Further exploration of the GEPIA database revealed that CD47, CTSZ, and SLC11A1 were highly expressed in various cancers, such as glioma, acute myeloid leukemia-like tumors, and pancreatic cancer (Fig. 8A–C). Therefore, we explored the expression of these three genes in both glioma cell lines and glioma patient tissues using the Human Protein Atlas database. Our findings demonstrated that these three genes were significantly upregulated in glioma cell lines, including LN229, U118MG, and other glioma cell lines (Fig. 8D–F), and were also found to be overexpressed in glioma patients (Fig. 8G–I). In glioma patients from the TCGA database, high expression of these key genes was associated with a poor prognosis (Fig. 8J–L). These analyses indicate that these key genes have strong cancer characteristics.

## 4 Discussion

Glioma, the most prevalent primary malignant intracranial tumor, poses substantial challenges due to its poor prognosis, high disability rate, and frequent recurrence, thereby imposing a significant burden on patients, families, and society. Among gliomas, glioblastoma stands out for its extreme malignancy. Despite aggressive treatment combining surgical resection and temozolomide-based chemoradiotherapy, the median survival time remains less than 15 months. Unlike tumors in other organs, gliomas display complex characteristics. Malignant glioma cells exhibit robust invasive abilities and infiltrate normal tissues early in tumor formation. Consequently, complete surgical resection often fails to prevent recurrence at surgical margins[[25], [26], [27]].

Tumor development and progression typically involve abnormal activation of proto-oncogenes and the loss or suppression of tumor suppressor genes. As tumors evolve, their microenvironment undergoes changes, including hypoxia, interstitial hypertension, and inflammatory reactive immunosuppression, collectively fostering continuous tumor growth and spread. This microenvironmental adaptation critically influences tumor behavior, treatment response, and patient prognosis. Advances in next-generation sequencing and molecular databases have revolutionized, enhancing gene sequencing efficiency and accuracy while providing extensive molecular data resources. The 2021 update by the World Health Organization on Glioma Neuropathology and Molecular Pathology Diagnostics underscores the need for refined molecular classifications guiding clinical interventions and prognostic stratification.

In our study, single-cell analysis facilitated the molecular classification of gliomas. Two distinct subtypes were identified using the NMF method. The GMC-I subtype, which is associated with poor prognosis, exhibited increased infiltration of high-grade gliomas in our sample cohort. Additionally, enrichment was observed among older patients, those with 1p19q non-codeleted status, those with *MGMT*p_Un-methylated status, and those with wild-type IDH within the GMC-I subtype. Differential gene expression analysis highlighted the activation of tumor proliferation-related and immune-related pathways, as well as associations with various tumors, including high-grade gliomas. Surprisingly, the GMC-I subtype showed abnormal activation of EMT [28], activation of the P53 pathway [29,30], activation of IL6-JAK-STAT3 signaling, and activation of multiple tumorigenic pathways. In contrast, the GMC-NI subpopulation exhibited elevated immune cell infiltration (T cells, B cells, and NK cells), which is indicative of an immune-activated state. These findings suggest that glioma patients with characteristics of the GMC-NI subtype are more likely to have “hot immune tumors”. Conversely, fibroblasts and neutrophils predominated in the GMC-I subtype, with significant enrichment of molecular markers associated with immune suppression and rejection, such as the EMT pathway, and were also significantly enriched in the GMC-I group, indicating an immune-suppressive state. Thus, the GMC-I subtype tends to exhibit a “cold immune tumor” phenotype, with M2 macrophage infiltration correlating with poorer survival outcomes in glioma patients [31,32].

To more accurately quantify glioma subtypes and identify glioma molecular markers, we employed various advanced analytical methods, including differential analysis, WGCNA, circular survival analysis, multiple machine learning algorithms, and protein–protein interaction (PPI) analysis. These multitiered analytical strategies facilitated comprehensive data exploration and culminated in the identification of key genes (CD47, CTSZ, and SLC11A1) pivotal for understanding the prognosis of the two subtypes. Rigorous validation across multiple independent datasets ensured the robustness and reproducibility of our findings.

In recent years, with the continuous innovation in therapeutic technologies, researchers have actively explored and applied advanced therapies for innovative treatment of various types of tumors. Traditional immunotherapies have mostly focused on stimulating the function of the adaptive immune system, particularly T cells. Among these, CD47, a highly N-glycosylated 47–52 kD integral membrane glycoprotein, is overexpressed in numerous cancer types and is expressed in almost all normal cells [33], including red blood cells (RBCs) and platelets. Notably, the high expression of CD47 in various tumor cells makes it a hallmark biomarker for malignant tumors [34]. As an immune checkpoint in the myeloid-specific system, CD47 effectively protects cancer cells from phagocytosis by binding to SIRPα on phagocytic cells, thereby inhibiting the phagocytic activity of macrophages in the immune system[33,[35], [36], [37], [38]]. Discovered as the first tumor phagocytosis checkpoint at the end of the 21st century, blocking the CD47-SIRPα axis or combining it with other therapies has been proven to be a promising treatment strategy in cancer immunotherapy. Clinical trial results have shown that targeted therapy against CD47 has achieved significant efficacy in various malignant hematological diseases [39,40] and solid tumors, making CD47 targeting a potential turning point in cancer immunotherapy [38]. Our study further reveals that CD47 can serve as a biomarker for patients with the GMC-I subtype of glioma, and modulating CD47 molecules may reverse the poor prognosis of the GMC-I subtype, providing guidance for clinical treatment. Meanwhile, the insensitivity of the GMC-I subtype to immunotherapy may be closely related to the overexpression of CD47. In addition, our study also found that CTSZ and SLC11A1 are diagnostic and prognostic biomarkers for patients with the GMC-I subtype, which is consistent with previous research [41]. Studies have shown that the immune-oncology-related gene SLC11A1 is overexpressed in high-grade gliomas and may be a biomarker for predicting the response of high-grade gliomas to temozolomide (TMZ) treatment. These targets demonstrate great potential in immunotherapy, and the overexpression of immune-oncology-related genes such as NFSF14, LY96, SLC11A1, and CTSL is associated with shorter survival in glioblastoma patients, serving as stratification indicators for immunotherapy or chemotherapy in glioma patients [42]. It is worth mentioning that SLC11A1 is also a potential biomarker for the prognosis and immunotherapy response in colorectal cancer [43]. Proteases play a crucial role in regulating various tumorigenic processes [44], including angiogenesis, tumor growth, and invasion. Elevated protease expression is closely related to poor prognosis in patients with various tumor types. Cathepsin Z (CtsZ) is a protease provided by both cancer cells and macrophages in human and mouse pancreatic neuroendocrine tumors. During tumor progression, cancer cells not only produce essential factors that promote growth and invasion but also rely on non-cancerous cells in the tumor microenvironment as alternative extracellular sources. The tumor-promoting function of CtsZ is mediated by the Arg-Gly-Asp (RGD) motif in its proenzyme domain, which regulates interactions with integrins and the extracellular matrix [45]. CtsZ provided by tumor-associated macrophages is identified as a compensatory protease that can regulate the acquired tumor-promoting function of lesions lacking CtsB and CtsS. Compared with normal glial cells, the expression of ALDH3B1 and CTSZ is significantly upregulated in glioma cells. In summary, this hypoxia-derived gene signature has the potential to be a reliable biomarker for predicting prognosis and treatment response, providing theoretical support for hypoxia-targeted therapy and precision care in glioblastoma (GBM) patients. In studies of clear cell renal cell carcinoma (KIRC) [46], remodeling the tumor microenvironment can promote its progression. Case-control studies have shown that changes in CTSZ methylation levels in peripheral blood may be associated with breast cancer, particularly in young women, and have the potential to be a potential biomarker for early breast cancer [47]. In addition, in lung cancer research, the combined loss of Ctsb and Ctsz has a cumulative effect, significantly delaying the development of both early and advanced tumors, improving histopathological tumor grade, and reducing the number of lung metastases by 70 % and the size of lung metastasis foci by 80 % [48]. The integration of the quantification results with the immune profiles of the two subtypes yielded cohesive insights. Systematic exploration of gene expression patterns across diverse tumor types, cell lines, and patient cohorts underscores the pivotal roles of identified key genes in tumorigenesis and prognosis. This comprehensive investigation provides foundational insights into molecular mechanisms across tumors, offering new avenues for further therapeutic exploration.

In summary, we developed a molecular classification approach based on cancer cell-related genes to enhance prognostic accuracy in glioma patients. By elucidating the clinical characteristics, molecular features, immune function, tumorigenic pathways, and the TME across the two distinct subtypes, our study advances the understanding of glioma molecular classification. In this regard, our study paves the way for new research directions in the molecular classification of glioma and holds significant scientific value. Future research should focus on unraveling the underlying mechanisms governing subtype-specific glioma behaviors, aiming to translate these insights into improved clinical strategies for diagnosis and treatment. Despite our progress, the precise mechanisms by which these relevant genes and subtypes influence glioma prognosis remain incompletely understood. Investigating the intrinsic mechanisms of these subtypes in glioma represents a major direction for future research. Future research should focus on unraveling the underlying mechanisms governing subtype-specific glioma behaviors, aiming to translate these insights into improved clinical strategies for diagnosis and treatment.

## CRediT authorship contribution statement

**Ying Dang:** Writing – review & editing, Writing – original draft. **Youhu Chen:** Methodology, Data curation. **Jie Chen:** Data curation. **Guoqiang Yuan:** Visualization, Supervision, Conceptualization. **Yawen Pan:** Visualization, Validation, Supervision, Resources, Methodology.

## Consent for publication

All authors have provided their final approval for the version to be published, have agreed to the submission of the article to the journal, and have consented to assume responsibility for all aspects of the work.

## Data availability

This study utilized publicly available datasets, which can be accessed from the following sources: The China Glioma Genome Atlas (CGGA) database (http://www.cgga.org.cn), The Cancer Genome Atlas (TCGA) database (https://portal.gdc.cancer.gov/).

## Declaration of generative AI and AI-assisted technologies in the writing process

During the preparation of this work, the author utilized Wenxin Yiyan and ChatGPT 3.5 to enhance the language quality and fluency of the manuscript. After the assistance of these intelligent tools, the author conducted a thorough review and made necessary editorial adjustments to ensure the accuracy, coherence, and originality of the content. The author hereby declares full responsibility for the content of the final publication, and all AI-assisted modifications have been appropriately revised and confirmed under the author's strict supervision.

## Funding statement

No funding was received for this research or the preparation of this article.

## Declaration of competing interest

We declare that we have no financial and personal relationships with other people or organizations that can inappropriately influence our work.

## Figures and Tables

**figs1:**
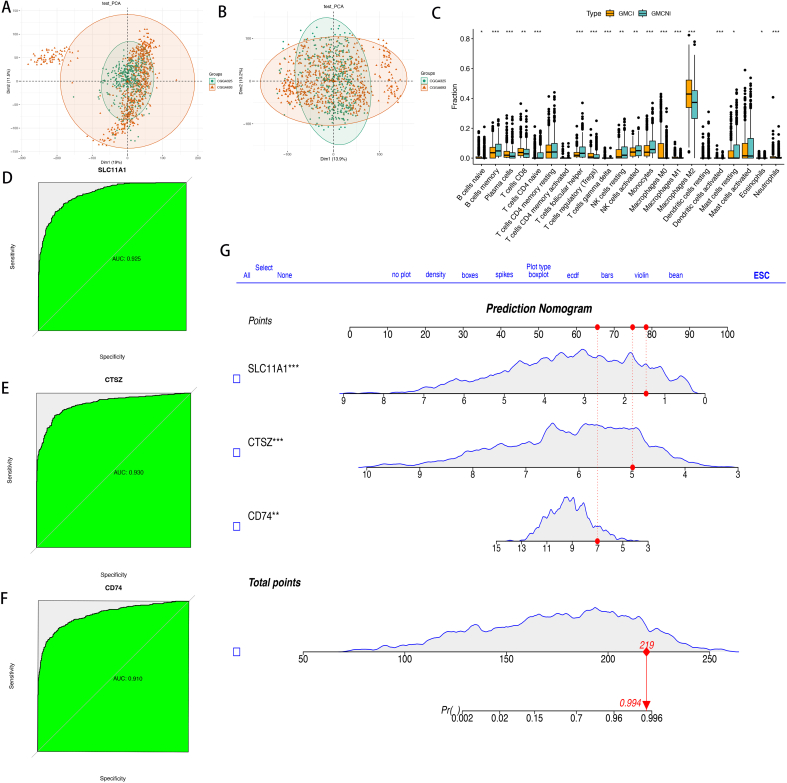


## Data Availability

Data will be made available on request.
